# Absence of M-Ras modulates social behavior in mice

**DOI:** 10.1186/s12868-015-0209-8

**Published:** 2015-10-21

**Authors:** Annette Ehrhardt, Bin Wang, Marie J. Leung, John W. Schrader

**Affiliations:** The Biomedical Research Centre, University of British Columbia, 2222 Health Sciences Mall, Vancouver, V6T 1Z3 Canada

**Keywords:** R-Ras3, Social recognition, VNO, Anxiety, Behavior heatmap

## Abstract

**Background:**

The molecular mechanisms that determine social behavior are poorly understood. Pheromones play a critical role in social recognition in most animals, including mice, but how these are converted into behavioral responses is largely unknown. Here, we report that the absence of the small GTPase M-Ras affects social behavior in mice.

**Results:**

In their interactions with other males, *Mras*^−*/*−^ males exhibited high levels of territorial aggression and social investigations, and increased fear-related behavior. They also showed increased mating behavior with females. Curiously, increased aggression and mating behaviors were only observed when *Mras*^−*/*−^ males were paired with *Mras*^−*/*−^ partners, but were significantly reduced when paired with wild-type (WT) mice. Since mice use pheromonal cues to identify other individuals, we explored the possibility that pheromone detection may be altered in *Mras*^−*/*−^ mice. Unlike WT mice, *Mras*^−*/*−^ did not show a preference for exploring unfamiliar urinary pheromones or unfamiliar isogenic mice. Although this could indicate that vomeronasal function and/or olfactory learning may be compromised in *Mras*^−*/*−^ mice, these observations were not fully consistent with the differential behavioral responses to WT and *Mras*^−*/*−^ interaction partners by *Mras*^−*/*−^ males. In addition, induction of *c*-*fos* upon pheromone exposure or in response to mating was similar in WT and *Mras*^−*/*−^ mice, as was the ex vivo expansion of neural progenitors with EGF. This indicated that acute pheromone detection and processing was likely intact. However, urinary metabolite profiles differed between *Mras*^−*/*−^ and WT males.

**Conclusions:**

The changes in behaviors displayed by *Mras*^−*/*−^ mice are likely due to a complex combination of factors that may include an inherent predisposition to increased aggression and sexual behavior, and the production of distinct pheromones that could override the preference for unfamiliar social odors. Olfactory and/or social learning processes may thus be compromised in *Mras*^−*/*−^ mice.

**Electronic supplementary material:**

The online version of this article (doi:10.1186/s12868-015-0209-8) contains supplementary material, which is available to authorized users.

## Background

Understanding the determinants that influence social behavior of animals has been a matter of numerous investigations, not least because of the potential to gain insight into human abnormal behavior. Both genetic and environmental factors, including epigenetics and learning experiences or cultural conditioning, shape complex behaviors and social interactions [1, 2]. Several genes have been identified that regulate social behavior of mice and other organisms [1, 3] and current research is focused on uncovering how these genes affect the molecular and neural mechanisms that underlie these behaviors.

Social interactions and the display of social behavior require recognition of an interaction partner. Mice, like most vertebrates, use olfactory cues (pheromones and kairomones) to identify other individuals of their own and other species. The successful identification then initiates an appropriate behavioral response. Pheromones convey information about kinship, gender, sexual receptivity, social status, or food toxicity, and therefore trigger innate social behaviors, ranging from parental care for offspring or aggression towards an intruder of their territory, to reproductive behaviors. Thus, pheromones regulate most aspects of social interactions between mice [4–6].

Both volatile and non-volatile substances, including proteins and large organic compounds, can act as pheromones [7]. In most animals, volatile pheromones are detected by G protein-coupled receptors in the two primary olfactory sensory organs, the main olfactory epithelium (MOE), and the vomeronasal organ (VNO) located at the bottom of the nasal cavity. The MOE is thought to facilitate associative olfactory learning and instinctive behaviors, while the VNO also detects non-volatile pheromones and is considered specialized for innate responses. Humans and some primates lack a functional VNO and may detect pheromones in the MOE (reviewed in [4, 8]).

The ability to recognize and to distinguish between familiar and unfamiliar individuals is the result of an olfactory learning process. While not yet well understood, this process involves specific pheromone activation profiles of neurons in the VNO [9] and neurogenesis in the subventricular zone (SVZ) [10, 11], and was shown to be supported by prolactin [10, 12, 13]. These factors contribute to the immediate recognition of an individual, an olfactory learning process, and the formation of an olfactory memory that determines future behavior [9–11, 14]. Estrogens and androgens, and the two closely related hormones oxytocin and vasopressin have been implicated in social recognition and/or social odor memory formation [15–17]. However, how pheromones are processed, once an opponent is recognized, to ultimately elicit specific behavioral responses is largely unknown.

We and others have previously characterized the small GTPase M-Ras/R-Ras3, which is closely related to members of the p21 Ras family of oncogenes (H-Ras, K-Ras and N-Ras) that are frequently mutated in many types of human cancers [18–22]. M-Ras may be overexpressed in some human cancers [23], but mutations overall are very rare and no activating mutations in the typical hot spots (G22, Q71) have been found in over 20,000 sequenced cancers (COSMIC database). Thus, its role in human cancers is presently unclear. Initial studies of mice lacking M-Ras did not reveal any obvious abnormalities [24]. We have serendipitously discovered changes in social behavior of *Mras*^−*/*−^ mice. We have described these changes and have explored some of the possible causes in the present study.

## Results

### Social and aggressive behavioral analysis of WT and *Mras*^−*/*−^ males

**Fig. 1:**
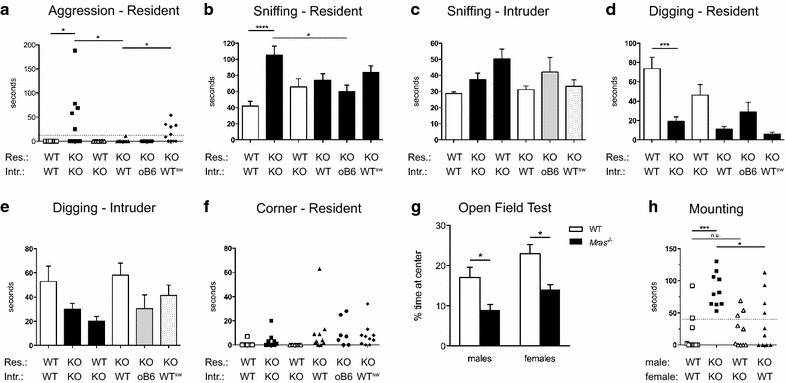
Altered social behavior in *Mras* ^−*/*−^ (KO) males. **a** Cumulative duration of aggressive behavior by the resident was scored during 10-min resident-intruder tests. *Mras* ^−*/*−^ (KO) residents displayed enhanced aggressive behavior towards KO and WT^sw^ intruders but not towards WT or oB6 intruders (‘oB6’: males that were on the C57BL/6 J background but that either had one knockout mutation or that expressed one transgene). WT/WT (res./intr.) vs. KO/KO, *p* = 0.0325; KO/KO vs. KO/WT, *p* = 0.0325; KO/KO vs. KO/oB6, *p* = 0.0441; KO/WT vs. KO/WT^sw^, *p* = 0.0325; KO/oB6 vs. KO/WT^sw^, *p* = 0.0441; all others not significant. **b** Social investigations (sniffing) by the residents during the first 5 min of the resident-intruder tests were scored. WT/WT (res./intr.) vs. KO/KO, *p* < 0.0001; KO/KO vs. KO/oB6, *p* = 0.0185. **c** Social investigations by the intruders during the first five min. of the resident-intruder tests were scored. There were no significant differences for both WT and KO intruders. **d** Digging behavior by the residents was scored during the first 5 min of the resident-intruder tests. *p* = 0.0001 for WT/WT vs. KO/KO. **e** Digging behavior by the intruders was scored during the first 5 min of the resident-intruder tests. There were no significant differences for both WT and KO intruders. **b**–**e** *Error bars* represent SEM. **f** ‘Corner’ behavior was scored for residents, showing more frequent anxiety-related behavior of *Mras* ^−*/*−^ (KO) males. *p* < 0.0001 for all WT residents vs. all KO residents combined; not significant between individual groups. **a**–**f** n = 10 for all groups except oB6, where n = 7. **g** Open Field tests: The amount of time mice spent at the center of an open and brightly lit white box was scored over a period of ten min. *Error bars* represent SEM, n = 10 for all groups except n = 11 for *Mras* ^−*/*−^ males; *p* = 0.0225; females, *p* = 0.0134. **h** Mating assays: Male mating behavior was scored in 10-min. interactions between combinations of WT and KO males and females. n = 10 for all groups; *p* = 0.0007 for WT/WT (male/female) vs. KO/KO; *p* = 0.0108 for KO/WT vs. KO/KO; not significant for WT/WT vs. WT/KO

High levels of aggression amongst the males in our *Mras*^−*/*−^ colony, especially after cage changes, prompted us to perform a formal analysis of social and aggressive behavior. We subjected *Mras*^−*/*−^ males to resident-intruder tests and compared their behavior to that of WT males. In addition, we exposed resident males to intruders of the opposite genotype to determine if this would affect interactions. We visualized the interactions by creating ‘behavior heatmaps’ of observable behaviors (aggression, social investigations, digging, ‘corner’, grooming) displayed by each mouse over time (Additional file 1: Figure S1A) and quantified these behaviors (Fig. 1a–f). There were striking differences between behaviors exhibited by WT and *Mras*^−*/*−^ males in their roles as residents.

When paired with intruders of their own genotype, half of the *Mras*^−*/*−^ resident males exhibited aggression (tail rattling, wrestling, biting, mounting, or lunging towards the intruder; Fig. 1a). Although the C57BL/6 J mouse strain is considered low to medium aggressive [25, 26], our WT males did not exhibit aggression during the brief 10 min. assessment period (Fig. 1a). The difference in aggressive behavior displayed by WT vs. *Mras*^−*/*−^ residents was significant (*p* = 0.0325). We also observed a significant increase in the rate of parental cannibalism of neonates in the *Mras*^−*/*−^ colony (*p* = 0.0062): WT breeder pairs (17 unique pairs) cannibalized 13 of 87 litters (14.9 %), whereas *Mras*^−*/*−^ breeder pairs (22 unique pairs) cannibalized twice as many litters (32 of 99, or 32.2 %). Cannibalism can be explained by parental aggression, although it can occur due to other factors, such as stress. During resident-intruder tests with isogenic male intruders, *Mras*^−*/*−^ residents also spent significantly more time investigating intruders compared to WT residents (F_5,51_ = 5.995, *p* = 0.0002; *p* < 0.0001; Fig. 1b). In contrast, WT residents exhibited significantly increased digging behavior compared to *Mras*^−*/*−^ residents (F_5,51_ = 10.76, *p* < 0.0001; *p* = 0.0001; Fig. 1d). Thus, in interactions with isogenic males, WT and *Mras*^−*/*−^ residents displayed social behaviors that differed significantly from one another.

Curiously, when *Mras*^−*/*−^ resident males were paired with WT intruders, aggression decreased significantly (*p* = 0.0325; Fig. 1a). *Mras*^−*/*−^ residents also spent less time investigating WT intruders less, although this did not reach significance (F_5,51_ = 5.995, *p* = 0.0002; *p* = 0.1343; Fig. 1b). In addition, *Mras*^−*/*−^ residents did not attack other knockout or transgenic mice on a C57BL/6 J background (but that expressed *Mras*; *p* = 0.0441) and displayed significantly decreased social investigations towards these intruders compared to *Mras*^−*/*−^ intruders (F_5,51_ = 5.995, *p* = 0.0002; *p* = 0.0185; Fig. 1a, b). In contrast, WT residents did not change their behavior when paired with *Mras*^−*/*−^ intruders (Fig. 1a, b). Thus, the expression of aggression by *Mras*^−*/*−^ males depended on the genotype of the intruder; *Mras*^−*/*−^ residents reacted aggressively to *Mras*^−*/*−^ intruder mice but not to mice that express M-Ras, while WT residents either did not make this distinction or did not react to it.

Mice use pheromonal cues to identify other individuals. Thus, we evaluated the behavior of *Mras*^−*/*−^ residents in the presence of WT intruders that were swabbed with urine from *Mras*^−*/*−^ males (WT^sw^). WT^sw^ intruders did not elicit aggression from WT residents (not shown), but elicited aggression from six out of ten *Mras*^−*/*−^ residents (60 %), which was similar to the frequency observed with *Mras*^−*/*−^ intruders (50 %; Fig. 1a). There difference in aggressive behavior of *Mras*^−*/*−^ residents towards WT vs. WT^sw^ intruders was significant (*p* = 0.0325). Aggressive behavior by *Mras*^−*/*−^ residents may not have been not fully restored with *Mras*^−*/*−^ social odors because attack duration was shorter on average (but not significantly shorter) with WT^sw^ intruders compared to *Mras*^−*/*−^ intruders (Fig. 1a). WT^sw^ intruders stimulated social investigations from *Mras*^−*/*−^ residents at levels that were similar to *Mras*^−*/*−^ intruders (Fig. 1b; Additional file 1: Figure S1A). These results suggested that *Mras*^−*/*−^ males may produce urinary pheromones that are distinct in quality or quantity from WT males and that could also contain elevated levels of aggression pheromones [14, 27]. However, *Mras*^−*/*−^ pheromones alone were not sufficient to elicit aggressive behavior because WT mice were not more aggressive towards *Mras*^−*/*−^ intruders than towards WT intruders (Fig. 1a). Thus, other changes must have occurred in *Mras*^−*/*−^ males to elicit the aggressive behavioral response.

WT residents tended to spend more time investigating *Mras*^−*/*−^ intruders than WT intruders and exhibited less digging behavior in the presence of an *Mras*^−*/*−^ intruder, although these differences were not statistically significant (Fig. 1b, d; Additional file 1: Figure S1A). In addition, sniffing and digging behavior displayed by WT intruders was irrespective of the genotype of the resident (Fig. 1c, e). Thus, WT males followed similar behavioral patterns with isogenic and congenic interaction partners both in their respective roles as residents and as intruders. In contrast, *Mras*^−*/*−^ males adapted their social behaviors depending on the genotype of the interaction partner when they were residents, but not when they were intruders.

### Inreased anxiety-like behavior in *Mras*^−*/*−^ mice

*Mras*^−*/*−^ residents frequently retreated to the corner or short edge of the test cage during the resident-intruder test (Fig. 1f; Additional file 1: Figure S1A). This ‘corner’ behavior was reminiscent of a fear response, similar to the ‘corner’ behavior observed in open field tests that has been considered anxiety-like behavior [28–30]. ‘Corner’ behavior was very rare with WT residents (*p* < 0.0001 for WT vs. *Mras*^−*/*−^ residents). Compared to WT mice, *Mras*^−*/*−^ mice also exhibited significantly increased anxiety-like behavior in an open field test (F_1,37_ = 19.54, *p* < 0.0001; *p* = 0.0225 for males; *p* = 0.0134 for females; Fig. 1g), including significantly more time spent in the corners of the open field (*p* = 0.0166; Additional file 1: Figure S1B). The ‘corner’ behavior by *Mras*^−*/*−^ males in the resident-intruder test was similar in the presence of intruders with different genotypes/odortypes (Fig. 1f). This suggests that enhanced anxiety-like responses may be a general characteristic of *Mras*^−*/*−^ males that, unlike sniffing and aggressive behaviors, was not adapted to different opponents.

### Interactions with females

We next investigated interactions between male and female mice. We observed a marked increase in male mounting activity among isogenic *Mras*^−*/*−^ mating pairs relative to isogenic WT mating pairs (*p* = 0.0007; Fig. 1h). Ten out of ten *Mras*^−*/*−^ males engaged in mating behavior within a 10 min. test period, whereas only four of ten WT males did. Moreover, all ten *Mras*^−*/*−^ males spent more than 50 s displaying mating behavior with *Mras*^−*/*−^ females, whereas only one of ten WT males showed mounting behavior for more than 50 s with WT females (Fig. 1h). This was likely not due to a general lack of motivation or problems exhibiting mating behavior, as WT and *Mras*^−*/*−^ males were equally effective at impregnating proestrus females when interactions were allowed to proceed over night (>90 % efficiency). To test the ability of *Mras*^−*/*−^ males to discriminate between WT and *Mras*^−*/*−^ females, females of the opposite genotype were paired with the males. Intriguingly, while WT males showed about the same amount of mounting behavior with WT and *Mras*^−*/*−^ females, *Mras*^−*/*−^ males exhibited significantly reduced mounting activity when paired with a WT female (*p* = 0.0108 for WT vs. *Mras*^−*/*−^ females paired with *Mras*^−*/*−^ males; Fig. 1h). This result suggests that the display of increased mounting behavior by *Mras*^−*/*−^ males, like the display of aggression, may depend on the genotype of the interaction partner. In addition, this also suggests that *Mras*^−*/*−^ females, like *Mras*^−*/*−^ males, may produce pheromones that are different from the pheromones secreted by WT females. However, these pheromones alone were not sufficient to elicit an increase in mating behavior from WT males, and the increased mounting activity was therefore like aggression a characteristic of *Mras*^−*/*−^ males.

Female mating behavior was also assessed. We did not observe lordosis in any of the females tested (not shown), which may have been due to the short duration of the assay. We noted that *Mras*^−*/*−^ females appeared to spent significantly more time investigating *Mras*^−*/*−^ males compared to WT females investigating WT males (F_3,36_ = 9.139, *p* = 0.0001; *p* = 0.0001; Additional file 1: Figure S1C). WT females also appeared to spend significantly more time investigating *Mras*^−*/*−^ males than WT males (*p* = 0.0022), while *Mras*^−*/*−^ females appeared to spend about the same amount of time investigating males of both genotypes (Additional file 1: Figure S1C). However, female responses were likely affected by male behavior (which was significantly different between WT and *Mras*^−*/*−^ males), making interpretation of female behavior difficult.

### Comparable levels of testosterone and sexually dimorphic genes in WT and *Mras*^−*/*−^ males

**Fig. 2:**
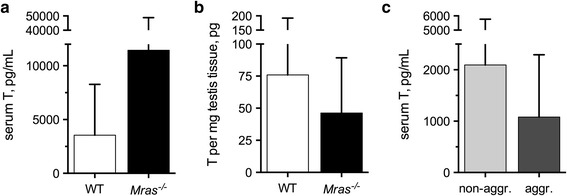
Testosterone levels. **a** Serum testosterone levels in WT and *Mras* ^−*/*−^ males (WT, n = 26; *Mras* ^−*/*−^, n = 31). **b** Total testosterone per mg testis tissue in WT and *Mras* ^−*/*−^ males (WT, n = 14; *Mras* ^−*/*−^, n = 19). **c** Serum testosterone in aggressive (n = 9) and non-aggressive (n = 10) *Mras* ^−*/*−^ males. All data is from six month-old males and shown with SD. **a**–**c** There were no significant differences in testosterone levels between WT and *Mras* ^−*/*−^ samples

Testosterone is related to aggressive behavior in animals [31, 32]. However, testosterone levels were comparable in the serum and testes of WT and *Mras*^−*/*−^ males, and those *Mras*^−*/*−^ residents that displayed territorial aggression did not exhibit significant changes in testosterone levels either (Fig. 2a–c). Levels of serum estradiol were also similar in WT and *Mras*^−*/*−^ males (Additional file 1: Figure S1D). Moreover, the expression levels of sexually dimorphic genes that have been implicated in male aggression and male and female sexual behavior [33, 34] were comparable in WT and *Mras*^−*/*−^ mice (Additional file 1: Figure S1E). These findings indicated that the lack of M-Ras caused changes in social behavior that depended on factors other than sex hormones, Sytl4, Irs4, Cckar, or progesterone receptor. They also indicated that *Mras*^−*/*−^ mice may secrete pheromones that differ from those of WT mice, and the possibility that *Mras*^−*/*−^ males may have an altered ability to sense pheromones and/or respond appropriately to pheromones.

### Lack of social odor discrimination by *Mras*^−*/*−^ mice

**Fig. 3:**
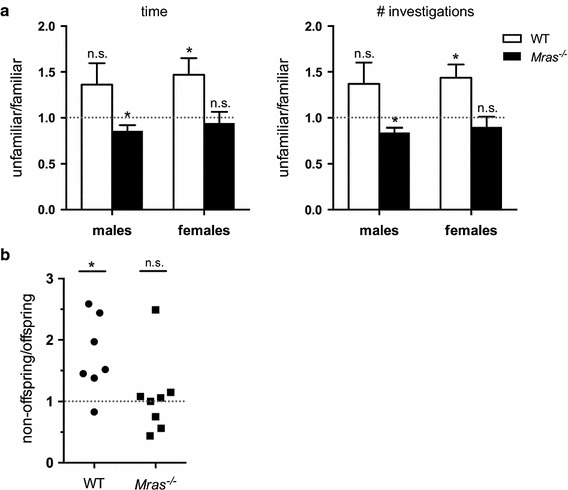
Evidence for defective vomeronasal function in *Mras* ^−*/*−^ mice. **a** Urine spot investigations. WT mice, but not *Mras* ^−*/*−^ mice, spent more time investigating an unfamiliar (congenic) over a familiar (isogenic) urine spot (*left panel*) and visited the unfamiliar spot more frequently (*right panel*). Data is shown with SEM; n = 11 for males, n = 12 for females; one-sample t-tests: WT females: *p* = 0.0263 (time), *p* = 0.0122 (# investigations); *Mras* ^−*/*−^ males: *p* = 0.0409 (time), *p* = 0.0107 (# investigations); all others, not significant. **b** Parental offspring recognition test. *Mras* ^−*/*−^ fathers, in contrast to WT fathers, were unable to discriminate between their own offspring and an unrelated isogenic mouse. One-sample t-tests: WT: *p* = 0.0205, n = 7; *Mras* ^−*/*−^: not significant, n = 8

Mice spend more time investigating unfamiliar scents over familiar ones [35, 36] and are able to distinguish between very subtle differences in pheromone compositions amongst genetically identical mice [9, 37]. We determined whether WT and *Mras*^−*/*−^ mice would discriminate between WT and *Mras*^−*/*−^ male urinary pheromones in the absence of the owner of the scent. As expected, WT females spent significantly greater time and frequency investigating the unfamiliar *Mras*^−*/*−^ male urine spots relative to WT urine spots (*p* = 0.0263 for time, *p* = 0.0122 for frequency; Fig. 3a). Similarly, WT males tended to spend more time investigating *Mras*^−*/*−^ urine spots, although this did not reach statistical significance (Fig. 3a). In contrast, both *Mras*^−*/*−^ males and females spent about the same amount of time investigating WT and *Mras*^−*/*−^ male urine spots, with *Mras*^−*/*−^ males exhibiting a slight but significant preference for *Mras*^−*/*−^ urine spots (*p* = 0.0404 for time, *p* = 0.0107 for frequency; Fig. 3a; also see Additional file 2: Figure S2A). We also tested the ability of WT and *Mras*^−*/*−^ fathers to recognize their own adult offspring. While WT fathers, as predicted, spent significantly more time investigating the alien isogenic offspring than their own offspring (*p* = 0.0205), *Mras*^−*/*−^ fathers spent the same amount of time investigating both their own and the alien isogenic offspring (Fig. 3b; also see Additional file 2: Figure S2B).

These results alluded to two possible underlying problems in *Mras*^−*/*−^ mice. That they were unable to recognize familiar pheromones or to discriminate between pheromones from isogenic and congenic mice suggests that pheromone detection, possibly by the vomeronasal organ (VNO), may be compromised in *Mras*^−*/*−^ mice. However, *Mras*^−*/*−^ males clearly displayed different behaviors with WT and *Mras*^−*/*−^ interaction partners (Fig. 1), which paradoxically indicated that pheromones were detected by *Mras*^−*/*−^ mice. Basic olfaction by the main olfactory bulb, as determined by a buried food test, and gross olfactory bulb anatomy were similar in WT and *Mras*^−*/*−^ males (Additional file 2: Figure S2C, D). Although immediate detection of pheromones may be intact, pheromone processing and subsequent olfactory learning could be defective in *Mras*^−*/*−^ mice. This may have rendered them unable to generate appropriate interpretations and social behaviors in response to pheromones. Alternatively, *Mras*^−*/*−^ males may produce distinct urinary pheromones that evoke increased interest in *Mras*^−*/*−^ mice (and possibly in WT mice). We addressed both possibilities in subsequent experiments.

### Known mechanisms that contribute to olfactory learning are similar in WT and *Mras*^−*/*−^ mice

**Fig. 4:**
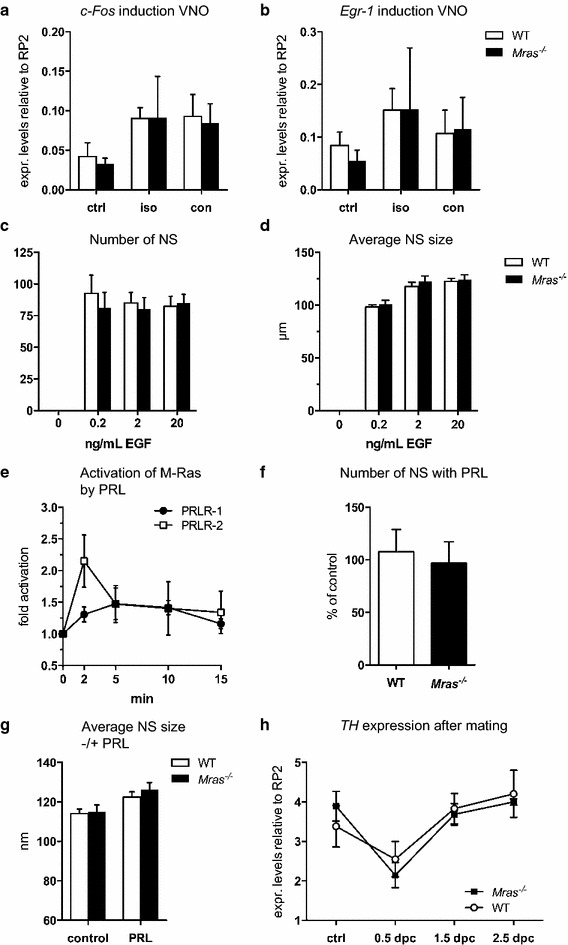
IEG induction, expansion of neural progenitors, and regulation of *TH*. **a**, **b** *c*-*Fos* and *Egr*-*1* gene induction in the VNO after exposure to alien male bedding for 30 min. Control (ctrl) mice remained in their own cage, ‘iso’ and ‘con’ refers to exposure to soiled bedding from isogenic or congenic mice, respectively. Data is shown with SD, n = 3 for all groups except *Mras* ^−*/*−^ exposed to ‘iso’, n = 5, and *Mras* ^−*/*−^ exposed to ‘con’, n = 4; no significant effect of genotype for either IEG. **c**, **d** The average number of NS generated (**c**) and average NS sizes (**d**) at varying concentrations of EGF were similar for WT and *Mras* ^−*/*−^ neural progenitors. There were no significant differences between WT and *Mras* ^−*/*−^ samples; n = 4 for experiments in **c**, n = 5 for experiments in **d**, *error bars* represent SD. **e** Stimulation of PRLR-1 or PRLR-2 with prolactin resulted in weak activation of M-Ras but with different kinetics; n = 5 for PRLR-1 samples (except t = 15 min, n = 4); n = 6 for PRLR-2 samples; *error bars* represent SEM. **f** Prolactin did not affect the number of NS generated (n = 4, error bars represent SD). **g** Prolactin slightly stimulated the proliferation of WT or *Mras* ^−*/*−^ neural progenitors (estimated by NS size) to similar levels; n = 184 WT ctrl, n = 198 WT + PRL, n = 127 *Mras* ^−*/*−^ ctrl, n = 123 *Mras* ^−*/*−^ + PRL; data shown with SEM. **h** *Tyrosine hydroxylase (TH)* gene expression in the olfactory bulbs of mated and plugged females (dpc: days post coitum). Data is shown with SD, n = 4 for all groups except *Mras* ^−*/*−^ 1.5 dpc where n = 3; no significant effect of genotype

We explored several processes that are known to affect olfactory learning to determine if they may underlie an apparent defect in VNO function in *Mras*^−*/*−^ mice. First, we tested acute pheromone sensing and signaling by G protein-coupled receptors in VNO neurons. Exposure to alien pheromones leads to the induction of the immediate-early genes (IEG), *c*-*Fos* and *Egr*-*1* [38], and Gα_o_ function in the VNO is required for aggressive behavior [39]). We exposed WT and *Mras*^−*/*−^ males to soiled bedding from either isogenic or congenic mice for 30 min. and analyzed IEG induction in VNO tissue. Exposure to either bedding source led to a significant upregulation of *c*-*Fos* (F_2,15_ = 5.724, *p* = 0.0142; Fig. 4a). *Egr*-*1* expression was also induced although not significantly (F_2,15_ = 2.308, *p* = 0.1337; Fig. 4b). There were no significant differences in the levels of induction of *c*-*Fos* and *Egr*-*1* between WT and *Mras*^−*/*−^ mice, which suggested that there were also no differences in G-protein coupled signaling by vomeronasal receptors leading to induction of these two key genes. Thus, acute pheromone sensing was likely intact in *Mras*^−*/*−^ mice, which supports our conclusion from the results shown above.

Epidermal growth factor (EGF) is the major growth factor driving the expansion of neural progenitor cells in the SVZ. As M-Ras is potently activated by EGF [24, 40] and is highly expressed in the brain, including the SVZ, the rostral-migratory stream, and the olfactory bulbs (see Additional file 3: Figure S3A, B, and Allen Brain Atlas), we evaluated EGF responsiveness of SVZ neural progenitors from WT and *Mras*^−*/*−^ mice in a neurosphere (NS) assay. However, the number of NS generated and their expansion with EGF, including long-term proliferation, was virtually identical in WT and *Mras*^−*/*−^ cells (Fig. 4c, d; Additional file 3: Figure S3C, D), which suggests that expansion of neural progenitors during olfactory learning is likely similar in WT and *Mras*^−*/*−^ mice.

Prolactin (PRL) acts through PRLR isoforms of the cytokine receptor family and so was predicted to stimulate M-Ras activation [40, 41]. Indeed, transcript variants PRLR-1 and PRLR-2 conferred weak but consistent M-Ras activation with different kinetics (F_2.278,6.834_ = 4.972, *p* = 0.0437 for PRLR1; F_2.101,10.51_ = 4.258, *p* = 0.0428 for PRLR2; Fig. 4e). PRLR-3, which has the shortest intracellular domain of the PRLR isoforms, did not activate M-Ras (not shown). The addition of prolactin to NS assays did not affect the number of NS generated from WT or *Mras*^−*/*−^ neural progenitors (Fig. 4f), but the average size of both WT and *Mras*^−*/*−^ NS increased slightly, and to a similar extent, with addition of prolactin to the cultures (F_1,628_ = 10.97, *p* = 0.0010; *p* = 0.4203; Fig. 4g, and see Additional file 3: Figure S3E), which indicated that prolactin mildly stimulated the proliferation of NSC. Interestingly, while SVZ tissue expressed detectable levels of all three PRLR isoforms, with the longest isoform PRLR-1 being the most abundant, we were unable to detect any PRLR transcripts in cultured NSC (see Additional file 3: Figure S3F). Thus, the proliferative stimulation likely occurred early in vitro, either before the neural progenitors downregulated PRLRs, or after supporting cells died off. Nevertheless, prolactin stimulated the expansion of neural progenitor cells to similar extents in both WT and *Mras*^−*/*−^ cells.

Another factor that can contribute to changes in social behavior is a change in motivation. Interest in mating and display of aggression can be considered motivation-based behaviors that are associated with reward, and both are associated with dopamine production or signaling [42–45]. Tyrosine hydroxylase (TH) is the key enzyme in dopamine biosynthesis; it can be used as an indirect measure of dopamine production [42]. We analyzed the changes in expression levels of *TH* upon mating in the olfactory bulbs of female mice. *TH* RNA levels changed significantly (F_3,23_ = 28.22, *p* < 0.0001), dropping early after mating (0.5 days post coitum) and subsequently increasing to steady-state levels or slightly above (Fig. 4h). The changes in *TH* expression levels were similar in WT and *Mras*^−*/*−^ mice, which suggests that dopamine metabolism was likely not significantly different between these mice.

### The urinary metabolite profile of *Mras*^−*/*−^ males may be distinct from WT males

**Fig. 5:**
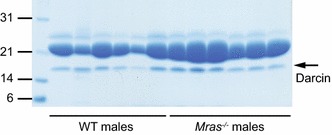
Major urinary proteins (MUPs) and Darcin. Urine was randomly collected from six WT and *Mras* ^−*/*−^ male mice each (ages: 2-5 months). Two µL of urine was loaded onto a 15 % SDS gel per sample. Gels were stained with Coomassie blue to visualize protein. MUPs are observed at 21 kD, Darcin at approximately 16-17 kD

Compounds that function as pheromones can be volatile or non-volatile chemicals, or can be proteins [5, 7]. To determine whether WT and *Mras*^−*/*−^ male urine differ in protein content, we tested randomly collected urine samples by SDS electrophoresis and Coomassie stain. The concentration of 21 kDa major urinary proteins (MUPs), which contain and may bind to pheromones, was slightly higher in *Mras*^−*/*−^ males (Fig. 5). However, the levels of Darcin (MUP20), which was identified as an attractive pheromone [46], were similar in WT and *Mras*^−*/*−^ males (Fig. 5). Next, we analyzed urine samples by NMR and mass spectrometry for urinary metabolites. Several metabolites were dysregulated in *Mras*^−*/*−^ urine (Table 1). For example, glycerate was increased 15.2-fold and succinate was decreased 4.8-fold. In addition, some components of lipid metabolism were detected at different levels in WT and *Mras*^−*/*−^ urine. Sixty-four percent of the phosphatidylcholines and 43 % of the lysophosphatidylcholines tested were increased at least two-fold, and 44 % of sphingomyelins were downregulated greater than two-fold in *Mras*^−*/*−^ urine compared to WT urine. Interestingly, *Mras*^−*/*−^ male urine contained twice as much trimethylamine as WT male urine. Trimethylamine has been identified as an attractive chemosignal in mouse urine [47]. The metabolomics analysis was performed only once with urine samples pooled from three males each and did not include many volatile compounds (and thus likely excluded some pheromones). However, the results from the resident-intruder tests may support the conclusion that the urinary metabolite/pheromone profiles likely differ between WT and *Mras*^−*/*−^ males, because *Mras*^−*/*−^ residents reacted differently to WT vs. WT^sw^ or *Mras*^−*/*−^ intruders (Fig. 1a, b).

**Table 1:** Urine metabolomics analysis

NMR	
Metabolite	Fold change in *Mras* ^−*/*−^ urine
Glycerate	+15.2
Methionine	+6.5
Ascorbate	+4.1
*N*-Carbamoyl-β-alanine	+3.9
*N*,*N*-Dimethylglycine	+3.1
Hypoxanthine	+2.8
Isoleucine	+2.6
Betaine	+2.6
Tyrosine	+2.5
2-Oxcisocaproate	+2.2
Trimethylamine	+2.0
Succinate	-4.8
1-Methlynicotinamide	−2.5
*N*-Phenylacetylglycine	−2.4
Hippurate	−2.2
Acetoacetate	−2.0

DI-MS			
Metabolite group	# Metabolites analyzed	Increased >2-fold in *Mras* ^−*/*−^ urine	Decreased >2-fold in *Mras* ^−*/*−^ urine
Acyl carnitines	40	1 (2.5 %)	1 (2.5 %)
Phosphatidylcholines	59	38 (64.4 %)	5 (8.5 %)
Lysophosphatidylcholines	7	3 (42.9 %)	1 (14.3 %)
Sphingomyelins	9	1 (11.1 %)	4 (44.4 %)

WT and *Mras* ^−*/*−^ male urine samples were analyzed by NMR and direct-injection mass spectrometry (DI-MS). Total number of metabolites analyzed by NMR: 65; DI-MS: 116. Some differences in relative metabolite quantities were confirmed by gas chromatography (GC)-MS analysis: Glyceric acid: +13.5; l-tyrosine: +2.41; succinic acid: −3.85; hippuric acid: −2.17 (fold-change in *Mras* ^−*/*−^ urine)

In summary, while none of the known factors contributing to pheromone sensing and olfactory learning appears to be compromised in *Mras*^−*/*−^ mice, it is possible that other, unexplored mechanisms may be affected by the absence of M-Ras. Moreover, it is likely that distinct pheromones produced by *Mras*^−*/*−^ males additionally affect their social behavior.

## Discussion

We uncovered changes in social behavior in mice lacking M-Ras, and used a graphical representation of dynamic social interactions, a behavior heatmap, to visualize differences in the ways WT and *Mras*^−*/*−^ mice dynamically interact with each other. *Mras*^−*/*−^ males exhibited higher levels of territorial aggression with other *Mras*^−*/*−^ males and spent more time than WT males to investigate an intruder; often, these prolonged investigations culminated in a physical attack. *Mras*^−*/*−^ mice displayed less digging behavior but increased fear-related responses such as the ‘corner’ response [28–30]. In mating assays with females, *Mras*^−*/*−^ males more readily engaged in mating behavior compared to their WT counterparts. Thus, *Mras*^−*/*−^ males showed social behaviors that were strikingly different from WT males.

The underlying causes for aggressive behavior have been the subject of intense investigations, particularly in humans. Although genetics and differentially regulated brain chemistry or neuronal circuits may play a role, aggression is also strongly influenced by experience (i.e. a learning process) and increases with positive reinforcement (reviewed in [48]). Another determinant of aggressive behavior is the interaction partner with his unique stimulus properties/characteristics [49–51]. Some mouse strains elicit higher levels of aggression than others, and hormone status and gender of the opponent can also affect aggressive responses [52, 53]. It is likely that olfactory (rather than visual and auditory) cues emanating from the opponents induce aggressive behaviors in resident males [54, 55].

Many different knockout mouse strains are known to exhibit changes in aggression levels [48, 56]. To our knowledge, our study is the first to show that an increase in aggressive behavior depended on two factors: (1) the absence of *Mras* in the resident and (2) the absence of a single gene in the intruder, *Mras* (as opposed to a difference in strain), unless compensatory changes in expression levels of other genes occurred during development. Our data supports that differentially secreted pheromones by *Mras*^−*/*−^ mice may contribute to the occurrence of aggression. If WT and *Mras*^−*/*−^ mice indeed emit different social odors, a WT intruder would be less familiar than an *Mras*^−*/*−^ intruder for an *Mras*^−*/*−^ resident male who had grown up in the presence of *Mras*^−*/*−^ but not WT social odors. Thus, the prediction was that the WT intruder should have suffered greater aggression [57]. That the opposite occurred was surprising and will be further discussed below. The increases in aggression and mounting activity in *Mras*^−*/*−^ males were unrelated to levels of testosterone or estradiol and expression levels of genes that are considered sexually dimorphic (Fig. 2; Additional file 1: Figure S1D, E). The serotonin system also contributes to the regulation of social interactions, including aggression, in humans and mice. Mice deficient in 5-HT_1B_ exhibit increases in aggressive behavior [58], and greater serotonin activity positively affects social interactions in humans and animals [59]. An optimal level of serotonin signaling is required during brain development for normal behavior in adulthood, and anxiety is associated with reduced function [60]. It will be of interest to determine if *Mras*^−*/*−^ mice exhibit changes in the serotonin system that could promote an increase in aggression and/or in fear-related behavior (Figs. 1a, f; Additional file 1: Figure S1B). Enhanced aggression and enhanced anxiety could contribute to an inability to appropriately assess contextual risk and adjust behavior accordingly.

WT and *Mras*^−*/*−^ resident males also differed in their digging behavior. Digging was a characteristic of WT but not *Mras*^−*/*−^ residents (Fig. 1d; Additional file 1: Figure S1A). Digging could represent an attempt to escape, perhaps in search of shelter [61]. We interpreted digging during the resident-intruder test as an expression of frustration (‘frustration digging’) [62]. Thus, it is possible that WT and *Mras*^−*/*−^ males may express frustration differently.

Another interesting observation emerged from our studies: *Mras*^−*/*−^ mice failed to discriminate both between highly similar, isogenic urinary pheromones (from their own offspring vs. other *Mras*^−*/*−^ mice), and even between pheromones from congenic and isogenic mice, where greater differences in pheromone composition would be expected (Fig. 3a, b). This could be interpreted as evidence for a defect in acute pheromone processing in *Mras*^−*/*−^ mice. The problems could be within the VNO and affect immediate detection. However, *c*-*fos* induction in the VNO in response to social odors was similar in WT and *Mras*^−*/*−^ males (Fig. 4a, b). Alternatively, processing of pheromone information downstream of the VNO may be defective. In particular, the medial amygdala integrates social odor cues from signals from both the main and accessory olfactory systems [63–65]. Although *Mras*^−*/*−^ mice did not discriminate between pheromones in the absence of the owners of these scents, they did discriminate between isogenic and congenic mice when the animal was present and available for social interactions, and exhibited enhanced aggression and mating behavior only towards isogenic partners (Fig. 1a, h). This seemed paradoxical, but suggested that pheromones were detected and that acute olfactory and/or vomeronasal function was intact in *Mras*^−*/*−^ males. There are several possible explanations.

First, independent of pheromones or their mechanisms of detection and processing, mice may be able to detect and interpret non-olfactory contextual information. This would depend both on the perception of the resident and on the behavior of the intruder. The intruder with his own characteristics will affect resident behavior [49–52]. For example, WT residents may detect differences in motor patterns or anxiety in *Mras*^−*/*−^ intruders and may not feel the need to attack, and *Mras*^−*/*−^ residents paired with *Mras*^−*/*−^ intruders could sense each other’s anxiety to produce changes in behavioral patterns [66, 67]. However, this is likely only a minor factor because *Mras*^−*/*−^ residents exhibited similar levels of aggression and social investigations with *Mras*^−*/*−^ intruders and WT intruders when the latter were swabbed with *Mras*^−*/*−^ urine, but they did not attack unswabbed WT intruders even though the WT intruders did not change observable behaviors whether they were swabbed with urine or not.

Second, we cannot rule out that the olfactory and/or social recognition processes may be malfunctioning in *Mras*^−*/*−^ mice, as we have only addressed a subset of molecular correlates. Immediate detection of pheromones was intact in *Mras*^−*/*−^ males, as assessed by the induction of *c*-*fos* upon pheromone exposure and the differential behavioral responses with WT and *Mras*^−*/*−^ territorial intruders (Figs. 1, 4a, b). In addition, neural progenitor cells of *Mras*^−*/*−^ mice had the same capacity to respond to an EGF or prolactin stimulus cells from WT mice (Fig. 4c–g). There are conceivably many more cellular and biochemical players that contribute to a highly complex learning process. For example, we have not addressed the possibility that *Mras*^−*/*−^ mice may exhibit epigenetic modifications, which could indicate that they inherited an olfactory experience from their ancestors [68]. Moreover, hormones such as oxytocin, vasopressin, and serotonin have been implicated in the molecular basis of social recognition. In particular, the closely related oxytocin and vasopressin may be involved in longer-term social recognition and modulation of behavioral responses in rodents [15, 69–71]. Mice lacking oxytocin do not display an increase in anxiety and a deficiency in the vasopressin receptor, V1aR, may result in male-specific reduction in anxiety levels [72–76]. In contrast, both male and female *Mras*^−*/*−^ mice showed higher levels of anxiety. Vasopressin may play dual roles in the regulation of aggressive behavior, facilitating or inhibiting aggression depending on the specific site of release in the brain [77–79]. It will be worthwhile to examine the contributions of these hormones to the behavioral changes in *Mras*^−*/*−^ mice. In addition, changes in other hormones and their receptors (e.g. corticosterone and brain estradiol; androgen and estrogen receptors) could drive the changes in social recognition and the associated differences in behavior.

Third, despite the evidence that points to a possible defect in VNO function, we cannot exclude a role for pheromones, or the processing of pheromones, in *Mras*^−*/*−^ mouse behavior. We observed that *Mras*^−*/*−^ mice may differentially produce several urinary metabolites compared to WT mice. We are currently exploring whether this may correlate with an enlargement of urinary bladders that frequently occurs in *Mras*^−*/*−^ males and seems to worsen with age (not shown here). One metabolite that was elevated in *Mras*^−*/*−^ urine, trimethylamine, is a known pheromone [47]. By virtue of its attractive properties, trimethylamine may facilitate social interactions between mice. Interestingly, high concentrations of trimethylamine can induce an aversive response in mice. The receptor for trimethylamine is TAAR [47]; it will be of interest to determine if TAAR levels are dysregulated in *Mras*^−*/*−^ mice. The altered secretion of trimethylamine and other urinary metabolites suggest that is very likely that the pheromone profile of *Mras*^−*/*−^ mice differs from WT mice. It was shown that mice that are deficient in oxytocin or estrogen receptor β produce urinary substances that facilitate an aversive response [80]. It is possible that *Mras*^−*/*−^ mice could produce distinct pheromones, such as trimethylamine, that may be perceived as enticing to both WT and *Mras*^−*/*−^ mice and affect behavioral responses. Darcin is another urinary protein that is perceived as attractive [46, 81], although its levels were similar in WT and *Mras*^−*/*−^ male urine (Fig. 5). The attraction of an *Mras*^−*/*−^ male to an as yet unidentified, *Mras*^−*/*−^-specific pheromone could cancel out the urge to explore a novel urine spot produced by a WT male when *Mras*^−*/*−^ urine is present at the same time, and could further stimulate a WT male to explore an *Mras*^−*/*−^ urine spot (Fig. 3a). Likewise, the unknown pheromone could restore exploration and attack of WT intruders by *Mras*^−*/*−^ residents when the former were swabbed with *Mras*^−*/*−^ urine (Fig. 1b), and could explain why *Mras*^−*/*−^ fathers investigate both their own and an alien offspring equally (Fig. 3b). The results from the offspring recognition tests, where test subjects were only exposed to airborne pheromones, suggest that the unknown, attractive pheromone could be volatile. Thus, the apparent defect in VNO function may be an artifact produced by an enticing pheromone secreted by *Mras*^−*/*−^ mice. However, even though urinary metabolites and pheromones may be differentially secreted by *Mras*^−*/*−^ mice, these alone were not sufficient to evoke an aggressive response or increased mating behavior by WT males, and additional mechanisms that could be involved with the processing of olfactory information must be dysregulated in *Mras*^−*/*−^ males for aggression and interest in mating to surface more readily. So far we have only explored the acute detection of pheromones in the VNO (by signaling through G protein-coupled receptors leading to the induction of *c*-*fos;* Fig. 4a, b) and the expansion of neural progenitors (Fig. 4c–f) and found that these were similar in WT and *Mras*^−*/*−^ mice. However, other molecular pathways downstream of the induction of IEGs, and/or as yet unknown molecular mechanisms and neural circuits that contribute to the processing of olfactory information could be affected by the absence of M-Ras.

Fourth, in combination with genetics and pheromones, learning will also affect behavior. Without a learning process, memory cannot be established. Interestingly, behavioral performance of neonates in an associative olfactory learning task was shown to depend on transcript levels of *Rasgrf1*, which is an activator of M-Ras [82, 83]. In addition, the *Mras* gene is upregulated upon spatial discrimination learning in rats [84]. We observed that *Mras*^−*/*−^ mice show a slight delay in spatial learning in the Morris water maze test, which was likely not due to an impairment in motor function. However, they caught up after eight training sessions and the consolidation of reference memory was similar to WT mice (see Additional file 4: Figures S4A-C). If *Mras* needs to be upregulated for an optimal hippocampus-dependent spatial learning task, it may also need to be increased for optimal olfaction- or pheromone-dependent social recognition, and the behavioral changes we have observed in *Mras*^−*/*−^ mice could be indicative of a slight learning deficit in general. Thus, *Mras*^−*/*−^ males could take longer to process pheromone information emitted by unfamiliar WT intruders and we cannot rule out that *Mras*^−*/*−^ resident males, when given more time, might attack WT intruders eventually. Likewise, *Mras*^−*/*−^ fathers could take longer to process the similar pheromones produced by their own and alien isogenic offspring, and may require more time to determine that there are differences. Alternatively, it is also possible that there may be defects in the formation of social odor memory. The oxytocin and vasopressin systems have roles in social odor memory consolidation [15], and more experiments are required to address the possibility of their involvement and to distinguish between short-term recognition (and actual recognition of an individual versus an ability to discriminate between familiar and unfamiliar opponents) and long-term memory formation. In addition, *Mras*^−*/*−^ pups could be fostered to WT parents in order to test whether there are problems with olfactory recognition or if there might be a component of faulty experiential learning through a parenting defect.

Finally, it is possible that the observed behavioral changes in *Mras*^−*/*−^ males could have been a consequence of ontogenetic processes caused by the deletion of the *Mras* gene, resulting in changes in expression levels of other genes. In addition, given the similarity to other proteins of the Ras family of GTPases, especially R-Ras, TC21, and the p21 Ras proteins, it is conceivable that these proteins could have taken over some of the normal functions of M-Ras, but could have skewed signaling pathways through slightly different effector usage [85]. Roles for Ras proteins in social behavior have not yet been reported, although neurofibromatosis 1 (Nf1), which inactivates Ras proteins by stimulating their GTPase activities, has been implicated in social recognition in mice [86]. It will be of interest to determine whether mice with deletions of other Ras proteins exhibit behavioral changes; this could provide insight into possible compensatory mechanisms in *Mras*^−*/*−^ mice.

## Conclusions

In summary, *Mras*^−*/*−^ males exhibited changes in social behavior that included increased territorial aggression, increased mating behavior, and fear-like responses. These changes were likely caused by a combination of several factors. Pheromones may be differentially produced and/or differentially sensed and interpreted by *Mras*^−*/*−^ mice. These pheromones could interfere with the correct identification of other individuals, which in turn could affect olfactory learning and result in an inability to respond appropriately to social stimuli. However, the behavior changes in *Mras*^−*/*−^ males cannot be explained by differences in pheromones alone, as these were not sufficient to elicit aggression or enhanced mating behavior from WT males. Other mechanisms, perhaps a signal transduction pathway that is under- or overactive by the absence of M-Ras must be altered to predispose *Mras*^−*/*−^ mice to display altered behavior. As social interactions in higher organisms are highly complex this will require more detailed investigations into the molecular pathways and neural circuits that M-Ras may affect.

## Methods

### Mice

*Mras*^−*/*−^ (KO) mice were obtained via contract from Lexicon Genetics and backcrossed to a C57BL/6 J (WT) background to F10. *Mras*^−*/*−^ mice on a mixed genetic background were previously reported to show no gross abnormalities [24]. We confirmed these observations after ten backcrosses. The lack of M-Ras protein expression was confirmed by Western blot (see Additional file 3: Figure S3B). *Mras*^−*/*−^ mice appear normal, fertile, and produce similar size litters as WT mice. Mice were generally group-housed at 2–5 mice per cage in yellow tinted plastic cages (30.5 × 12 × 14 cm) that contained structural enrichment (nesting material and a plastic toy/hiding place). A few aggressive *Mras*^−*/*−^ males were individually housed to prevent injury to their siblings. One of the aggressive *Mras*^−*/*−^ males was used in a resident-intruder test with a WT intruder; he did not exhibit aggression towards this intruder. Other aggressive, individually-housed *Mras*^−*/*−^ males were not used in tests that involved interactions with other mice. Mice were housed in a controlled environment with 12-h light/dark cycles (lights on at 6 am and off at 6 pm) and access to water and standard rodent chow (LabDiet, 50/50 mix of #5053 and #5058) ad libitum. The lack of M-Ras may predispose mice to the development of obesity: Old (6–12 months) *Mras*^−*/*−^ females raised on a regular diet were slightly (~5 g) heavier than WT females and somewhat resistant to weight loss after overnight fasting (not shown).

All mice were reared in the presence of both parents. Behavioral tests, with exception of the mating assays, were conducted during the light phase. Mice were not used for more than one behavioral examination and were only used once during the one behavioral test, with exception of a few mice that participated in the resident-intruder test, and those assessed in water maze (see below). Other strains used for the resident-intruder test: *Psgl1*^−*/*−^ mice (B6.Cg-Selplg^tm1Fur^/J; stock number: 004201; C57BL/6 J background) and *Wnt1*-*cre* mice (Tg[Wnt1-cre]11Rth Tg[Wnt1-GAL4]11Rth/J; stock number: 003829; backcrossed to C57BL/6 J in-house to F3) were purchased from The Jackson Laboratory. *Cd34*^−*/*−^ mice had originally been provided by Dr. T. Mak and were extensively backcrossed (>F10) in-house to C57BL/6 J. All three strains were gifts of other investigators at The University of British Columbia (Drs. H. Ziltener, F. Rossi, and K. McNagny). Urine collection: Urination was stimulated by having an experimenter that was unfamiliar to the mice place them onto a wire cage lid where they were gently held back by their bodies and tails as they tried to move forward. Immediately after collection, urine samples were frozen on dry ice and then stored at −80 °C for later uses. We followed general guidelines set forth by the Canadian Council on Animal Care. Animal protocols were approved by the Animal Care Committee of The University of British Columbia (Protocol Numbers: A08-0202 and A13-0213).

### Resident-intruder paradigm

Male mice (n = 10 per group) were tested for aggressive behaviors in a resident-intruder test at 6 months of age. This age was chosen because we noted slightly higher levels of aggression at this age than at 3 months; by 12 months of age intruder aggression became a problem. (Females were not tested because we have not observed aggressive behavior amongst *Mras*^−*/*−^ females during regular housing.) Each resident male was singly housed for 8 days and then tested in his home cage against a group-housed male intruder for 10 min. The intruders were not littermates of the residents and were never housed with the residents. The intruders were always younger than the residents, their ages ranged from three to nearly 6 months.

We tested several different combinations of resident/intruder pairs. WT residents were tested with WT or *Mras*^−*/*−^ intruders. *Mras*^−*/*−^ residents were tested with *Mras*^−*/*−^ and WT intruders. Three *Cd34*^−*/*−^, two *Psgl1*^−*/*−^, and two *Wnt1*-*Cre* transgenic mice, all on the C57BL/6 J background, were also used as intruders (‘oB6’) with one group of *Mras*^−*/*−^ residents. In addition, we tested *Mras*^−*/*−^ residents with WT intruders that had been swabbed with urine from *Mras*^−*/*−^ males immediately prior to testing (‘WT^sw^’). The urine had been collected earlier and stored at −80 °C. The urine samples originated from four different *Mras*^−*/*−^ males and were used randomly. In some cases one urine sample was used on several WT intruders; this did not lead to consistent aggression (or the lack thereof) by the residents. A total of 40 µL of urine was spread onto the backs and anogenital regions of the intruders. To test the effect of swabbing we presented six WT residents with WT intruders that had been swabbed with WT urine. There were no significant differences in sniffing or digging compared to WT residents that encountered unswabbed WT intruders, and aggressive behavior was not elicited. Some males were used twice in the resident-intruder tests: One WT male was used twice as an intruder with *Mras*^−*/*−^ residents, 1 day apart; one *Mras*^−*/*−^ male was used twice as an intruder with a WT and an *Mras*^−*/*−^ resident, 2 weeks apart; three *Mras*^−*/*−^ males were used as intruders with WT residents first and as residents with *Mras*^−*/*−^ intruders 2 weeks later; one *Mras*^−*/*−^ male was used as an intruder with an *Mras*^−*/*−^ resident first and 2 weeks later, as a resident, with a WT^sw^ intruder. Thus, we ensured that the WT social odors were novel for *Mras*^−*/*−^ residents in all cases.

Resident-intruder interactions were video-recorded and the cumulative duration of aggressive behavior was scored by visual inspection of the videos. Aggressive behavior was defined as tail rattling, wrestling, biting, mounting, or lunging towards the intruder. Non-aggressive behaviors such as social investigations (‘sniffing’), digging, grooming, and ‘corner’ (retreat to the corner or short edge of the cage and sitting still and/or crouching without engaging in other activities) were scored during the first 5 min of this test. The intruders hardly ever exhibited ‘corner’ behavior (1/57 pairs; not shown). The ‘corner’ behavior has been interpreted as anxiety-related behavior in open field tests [28–30]. ‘Sniffing’ scores may have been underestimated when aggression occurred. A ‘behavior heatmap’ was generated by breaking down the first 5 min of each mouse’s session into one-second intervals and by assigning each second a designated color for one of the five scored behaviors. The behavior heatmap is thus a graphical representation of all scored behaviors for each mouse over time.

### Anxiety-like behavior

Three month-old male and female mice were used for open field tests (n = 10 per group except n = 11 for *Mras*^−*/*−^ males). Tests were conducted in a white box, 59 × 59 cm. A square center was defined 15 cm from edges of the box, and the time spent in the center area (at least two paws inside) was scored during a 10 min testing period. Also scored was the time spent in the four corners of the apparatus (mouse within approximately one body length of the corner). The data was analyzed by two-way ANOVA.

### Mating assay

Estrus cycles were determined in female mice by vaginal swabbing and cytology; an abundance of epithelial cells with some nucleated cornified cells was considered proestrus. Proestrus females were placed in a clean test cage 2–4 h after lights-off. Males were added for 10 min and interactions between the mice were video-recorded. Mating behavior by the male (mounting or mounting attempts, thrusting) was scored. Female behavior was not scored because it was likely influenced by male behavior, which differed significantly between WT and *Mras*^−*/*−^ males. All mice were approximately 3 months old. Group size: 10 pairs per group.

### Urine spot investigations

Ten µL of urine from a WT or an *Mras*^−*/*−^ male was soaked into the ends of two cotton swabs and the swabs were placed at opposite ends of a clean test cage. Urine from isogenic mice (the “familiar” pheromone sample) did not originate from a cagemate of the test mouse. Mice (11 males and 12 females per group; 3–7 months old) were added to the test cage and video-recorded over a 10 min period. The time spent investigating either swab was scored, as was the frequency with which the swabs were visited. We determined ratios of unfamiliar/familiar investigations because mice differed in their activity levels; however, total times and latencies to investigate are shown in Additional file 2: Figure S2A. A one-sample *t* test was performed to determine whether the ratio of unfamiliar/familiar (time or number of investigations) was significantly different from 1.

### Parental offspring recognition

A three-chambered plexiglass box with perforated dividers was used for this test, as described by Mak et al. [37]. The two dividers each consisted of two thin, perforated metal sheets spaced 1 cm apart that prevented direct physical contact between mice but allowed for odors to pass through and for some limited visuals. One of the outer chambers contained a ~2 month old male offspring of the mouse father to be tested along with some of the bedding from his cage, the other outer chamber contained an age-matched male of the same genotype but sired by a different male, along with some of his bedding. The mouse father, who was continuously co-housed with his female mate and was present throughout pregnancy and rearing of their offspring, was placed in the middle chamber. Offspring had separated from their parents at 3 weeks of age, which means that mice had been separated for 5–6 weeks at the time of testing. Seven WT and eight *Mras*^−*/*−^ fathers with offspring were filmed for a period of five min. The amount of time the father spent in close proximity to the dividers on either side was scored (nose within ~0.5 cm or closer). A one-sample t-test was performed to determine whether the ratio of unfamiliar/familiar (time spent investigating, or frequency of investigations) was significantly different from 1. Total times and latencies to investigate are shown in Additional file 2: Figure S2B.

### Testosterone and estradiol levels

Blood was collected from 6 month-old male mice by cardiac puncture, left at room temperature for ~15 min., and centrifuged. Serum was stored at −20 °C. Serum testosterone and estradiol from *Mras*^−*/*−^ males was measured using EIA kits (Cayman Chemical). Intratesticular testosterone was extracted from whole testes of 6 month-old male mice. Testes were first mechanically lysed using small glass tissue grinders in 500 µL of RIPA buffer (150 mM NaCl, 50 mM Tris–HCl pH 8.0, 1 % NP-40, 0.5 % Na-deoxycholate, 0.1 % SDS) plus protease inhibitors (Roche). Testosterone was extracted from the tissue lysates with three successive extractions with 2.5 mL ether each. The ether extract was dried and pellets resuspended in EIA buffer for analysis by the Testosterone EIA kit (Cayman). We determined serum testosterone levels both in male mice that had undergone the resident-intruder test on the same day, and in mice that had not. There were no significant differences in testosterone levels between these mice. Serum estradiol was measured in males that had not undergone behavioral testing. Serum testosterone, n = 26, WT; n = 31, *Mras*^−*/*−^. Total testosterone per mg testis tissue, n = 14, WT; n = 19, *Mras*^−*/*−^. Serum testosterone in aggressive and non-aggressive *Mras*^−*/*−^ males, n = 9 and n = 10, respectively. Serum estradiol, n = 12 each.

### Neurosphere (NS) assay

The subventricular zone (SVZ) was dissected from the brains of 8 week-old male and female mice (we did not observe significant differences in the capacity to form NS from male or female cells; n = 5 groups of 2–3 mice each). The tissue was dissociated with Papain (Worthington) and cells were plated in NeuroCult NSC Basal medium with Proliferation Supplement in the presence of varying concentrations of EGF (all from StemCell Technologies) at 3000 cells per 24-well. FGF was omitted because we found that its addition induced some adhesion of neural progenitor cells and relatively poor formation of spheres especially at lower concentrations of EGF. For experiments shown in Fig. 4f, g and Additional file 3: Figure S3E, 2 ng/mL prolactin (Peprotech or Sigma) was added to NS cultures at the time of seeding. The number of NS generated and their diameters were scored 7 days after plating.

### Gene expression

RNA expression levels of *c*-*fos* and *Egr1* were determined in the vomeronasal organs of WT and *Mras*^−*/*−^ male mice (ages: 9–20 weeks) that had been exposed to soiled bedding for 30 min (n = 3 for all groups except n = 4 or n = 5 for *Mras*^−*/*−^ males exposed to bedding from congenic or isogenic mice, respectively). The soiled bedding was provided in the home cages of WT or *Mras*^−*/*−^ males that had lived in these cages in groups of 3–5 mice for 5 days and that had been removed from these cages (along with structural enrichment and food) immediately before the test mouse was added. Test mice were not siblings of mice that inhabited the cages used for exposure. RNA expression levels of *Prlr* isoforms were analyzed in SVZ tissue and cultured NS, and those of sexually dimorphic genes (*Sytl4*, *Cckar*, *Irs4*, *progesterone receptor [PR]*) in the hypothalami of 10–26 week-old mice (n = 7 for all male samples, n = 5 for all female samples). *Sytl4* levels were determined in males, *Cckar* and *Irs4* in females, because *Sytl4* regulates male behaviors while *Cckar* and *Irs4* regulate female behaviors, and mice of the respective opposite sexes lacking either of these genes do not exhibit changes in sex-specific behaviors [33]. Although *PR* is often referred to as a sexually dimorphic gene, it seems to regulate both female and male sexual behaviors [87–89], which is why we determined levels of *PR* in both sexes. *Tyrosine hydroxylase (TH)* RNA was extracted from the olfactory bulbs of mated and plugged 2–4 month-old females 0.5, 1.5, or 2.5 dpc; control females were not exposed to males (n = 4 per group except *Mras*^−*/*−^ at 1.5 dpc, n = 3). RNA was extracted from the tissues using Trizol and converted to cDNA with Superscript (both from Invitrogen). Real-time PCR was performed using an ABI 7900HT instrument with *PolR2A(RpII)* as the reference gene. We found that *PolR2A* levels were very similar in all samples (and not different between WT and *Mras*^−*/*−^ tissues) and confirmed its usefulness as a reference gene [90]. Primers are listed in Additional file 5: Table S1.

### M-Ras activation by prolactin

We cloned the three transcript variants of murine *Prlr* and the *Jak2* gene from mouse brain cDNA and inserted genes into pCDNA3.1. HEK293 cells were transiently transfected with the *Prlr* isoforms along with *Jak2* and myc-tagged *Mras* [40]. Serum-starved cells were stimulated with 1 µg/mL sheep prolactin (Sigma) for up to 15 min. Cell lysates were subjected to a pull-down assay with GST-Nore-1 RBD as the bait for activated, GTP-loaded M-Ras [40] and samples analyzed by Western blot with anti-myc antibodies (Cell Signaling Technology). The signal intensity of bands on blots was measured using ImageJ. Prolactin stimulations were performed four times for PRLR1 and six times for PRLR2. Data was analyzed by repeated-measures ANOVA.

### Urine metabolomics

Urine analysis was conducted at The Metabolomics Innovation Centre (University of Alberta, Edmonton, Canada). Urine was collected from three 4–5 month old WT or *Mras*^−*/*−^ males each. Pooled samples were analyzed by NMR and direct injection-mass spectrometry (DI-MS).

### Data analysis

Statistical analyses were carried out using Graphpad Prism 6.0. Significance was considered at *p* < 0.05; *p < 0.05, **p < 0.01, ***p < 0.001, ****p < 0.0001. Unless indicated otherwise, analysis of significant differences was performed using two-tailed t-tests or two-way ANOVA followed by Tukey’s post hoc analysis. We report *p* values from t-tests. For ANOVA analyses, F and *p* values are reported for the output of interest followed by subsequent post hoc *p* values in the format (F_dFn,dFd_, *p*; *p*). Results from resident-intruder tests were analyzed as follows. In cases where the data was not normally distributed (Fig. 1a–aggression, f–corner, h–mounting) we performed Fisher’s exact tests on the number of animals that exhibited the behavior for a minimum period of time versus animals that did not exhibit the behavior or were below the cutoff. Cutoffs were set to 12 s for aggression (Fig. 1a) and to 40 s for mating behavior (Fig. 1h). For the ‘corner’ behavior (Fig. 1g), we compared all WT residents to all KO residents (irrespective of the type of intruder); comparisons between individual groups were not significant. For comparisons of sniffing and digging behaviors (Fig. 1b–e) we used one-way ANOVA followed by Tukey’s post hoc analysis. Cannibalism data was analyzed by Fisher’s exact test. Data from the urine spot investigation and offspring recognition tests were analyzed by one-sample t-test to determine if values were significantly different from 1. Activation of M-Ras by prolactin was analyzed by repeated-measures ANOVA.

### Supplemental methods and results

Supplemental methods and results can be found in Additional file 6.

## Additional files

10.1186/s12868-015-0209-8 **(A)** Behavior heatmaps. Graphical representations of the first five min. of the resident-intruder tests, showing different colors for different behaviors for each test subject in one-second intervals. WT^sw^ denotes a WT intruder that was swabbed with *Mras* ^−*/*−^ male urine; oB6: other knockout and transgenic mice on a C57BL/6 J background. **(B)** Corner time in the open field apparatus. The total time spent in the four corners of the open field arena was scored for WT and *Mras* ^−*/*−^ males during a 10 min. session. Data shown with SEM, n = 10 for WT, n = 11 for *Mras* ^−*/*−^; *p* = 0.0166. **(C)** Social investigations by females in the mating assay. Data shown with SEM, n = 10 for all groups; *p* = 0.0001 for WT vs. *Mras* ^−*/*−^ females with isogenic partners; *p* = 0.0022 for WT females paired with WT males vs. *Mras* ^−*/*−^ males. Note that female behavior presumably is affected by male mating behavior. This was significantly different between WT and *Mras* ^−*/*−^ males, making interpretation of this data difficult. **(D)** Serum estradiol levels. Estradiol levels were measured in sera from six month-old WT and *Mras* ^−*/*−^ males. Data shown with SEM, n = 12 for both groups. **(E)** Expression of sexually dimorphic genes. *Sytl4, Irs4, Cckar* and progesterone receptor gene expression in the hypothalami of WT and *Mras* ^−*/*−^ mice was analyzed by qRT-PCR. Data is shown with SD; n = 7 for all male samples, n = 5 for all female samples; no significant differences between any of the WT and *Mras* ^−*/*−^ samples.
10.1186/s12868-015-0209-8 **(A)** Urine spot investigations. Total times investigating, and latencies to investigate the familiar (isogenic) urine sample and the unfamiliar (congenic) urine sample were scored in the urine spot investigations test. The total times spent investigating the urine spots varied considerably for all mice, which may reflect differences in activity levels (no significant differences). The latency times show that WT and *Mras* ^−*/*−^ males and females did not exhibit a preference for investigating a particular urine sample first. Females: n = 12 each; males: n = 11 each. **(B)** Parental offspring recognition. Total times investigating, and latencies to investigate the familiar (own) offspring and the unfamiliar isogenic offspring were scored in the parental offspring recognition test. The data shows that 5/7 (71 %) WT fathers investigated the unrelated offspring first while only 4/8 *Mras* ^−*/*−^ (50 %) fathers did. Also, 5/7 (71 %) WT fathers spent more total time investigating the unrelated offspring while only 1/8 (12.5 %) *Mras* ^−*/*−^ fathers spent greater time investigating the unrelated offspring (all others spent about the same time investigating either offspring, or spent more time investigating the familiar offspring). However, there were no significant differences between WT and *Mras* ^−*/*−^ fathers for either total time or latency (WT: n = 7; *Mras* ^−*/*−^: n = 8). **(C)** Basic olfaction. WT and *Mras* ^−*/*−^ mice (males, 3-6 months old) took similar amounts of time to detect the scent of a piece of novel food buried in bedding. Data is shown with SEM, WT: n = 10; *Mras* ^−*/*−^: n = 9; t-test: not significant. **(D**) Olfactory bulb anatomy. Frozen tissue sections of olfactory bulbs from three month-old WT and *Mras* ^−*/*−^ males were stained with Nissl. AOB: accessory olfactory bulb; SEZ: subendothelial zone; AON: accessory olfactory nerve.
10.1186/s12868-015-0209-8 **(A, B)** M-Ras protein expression in mouse tissues. **(A)** M-Ras expression was very high in brain (cortex), low in bladder, kidney, lung, spleen, and thymus, and undetectable in other tissues, including heart and skeletal muscle. **(B)** M-Ras protein expression in mouse brain regions. This Western blot shows highest expression of M-Ras in the cerebral cortex (COR) and lower expression in hypothalamus (HYP), hippocampus (HIP), and in the olfactory bulbs (OB). Note the absence of M-Ras in *Mras* ^−*/*−^ (KO) samples. **(C)** NS size frequency distributions at varying concentrations of EGF. Neural progenitor cells from SVZ tissue were cultured in the presence of different concentrations of EGF, as indicated. NS diameters (in µm) were measured at DIV7 (n = 6 for 0.2 ng/mL EGF, n = 5 for 2 ng/mL EGF, n = 3 for 20 ng/mL EGF; error bars represent SD). **(D)** Secondary NS growth. NSC from DIV7 NS grown in 2 ng/mL EGF were dissociated and replated at varying concentrations of EGF. To estimate cell proliferation, metabolic activity was measured six days after seeding by WST-1 assay (n = 3 for both WT and *Mras* ^−*/*−^; error bars represent SD; significant effect of EGF stimulation on growth (F_4,20_ = 90.42, *p* < 0.0001); no significant effect of genotype. **(E)** NS size frequency distribution with and without PRL. Neural progenitor cells were cultured in the presence of 2 ng/mL EGF and with or without 2 ng/mL sheep PRL (sPRL; left) or 2 ng/mL recombinant murine PRL (rmPRL; right). NS diameters were measured at DIV7. Virtually identical results were obtained with sPRL and rmPRL. **(F)** PRLR gene expression. Levels of the three transcript variants of PRLR were quantitated by real-time PCR in SVZ tissue and in NSC from DIV7 NS. No PRLR was detected in cultured primary NS (n = 3 for all samples; data shown with SD).
10.1186/s12868-015-0209-8 **(A)** Spatial learning in the water maze. *Mras* ^−*/*−^ mice showed a slight delay in a test for spatial learning, the Water Maze test, where they were scored for their ability to find a hidden platform in a pool of water. Both males (left) and females (right) were tested. Data is shown with SEM, n = 10 each; the effect of genotype was significant for males (F_1,290_ = 13.72, *p* = 0.0003; *p* = 0.0002 for WT vs. *Mras* ^−*/*−^ males on day 2), but not significant for females. For both males and females a significant interaction between genotype and trial day was detected. **(B)** Reference memory. Mice that underwent the Water Maze test received a probe trial the day after the last acquisition day. The time the mice spent swimming in the quadrant that had previously contained the hidden platform was scored during a 30 s session. WT and *Mras* ^−*/*−^ males and females performed similarly in this test (data is shown with SEM, n = 10 each, no significant differences). **(C)** Assessment of motor function. The floor of a 59x59 cm open field-like apparatus was divided into 36 squares. After two 15-min. habituation sessions in the apparatus over two days we determined how many squares the mice visited during a five minute session on the third day. There were no significant differences between WT and *Mras* ^−*/*−^ mice, or between male and female mice (n = 10 per group, data is shown with SEM).
10.1186/s12868-015-0209-8 List of primers used for qRT-PCR.
10.1186/s12868-015-0209-8 Supplemental Methods and Results.

## Abbreviations

AON: accessory olfactory nerve
COR: cerebral cortex
DI-MS: direct-injection mass spectrometry
dpc: days post coitum
EGF: epidermal growth factor
FGF: fibroblast growth factor
GTP: guanine triphosphate
HIP: hippocampus
HYP: hypothalamus
IEG: immediate-early gene
kDa: kilodalton
KO: knockout (here specifically of M-Ras)
MOE: main olfactory epithelium
MUP: major urinary protein
NMR: nuclear magnetic resonance
NS: neurospheres
NSC: neurosphere cells
OB: olfactory bulb
PolR2A/RpII: RNA polymerase II
PR: progesterone receptor
PRL: prolactin
PRLR: prolactin receptor
SEZ: subendothelial zone
SVZ: subventricular zone
TH: tyrosine hydroxylase
VNO: vomeronasal organ
WT: wild-type
