# Supplementary material for: Absence of M-Ras modulates social behavior in mice
Source: BMC Neurosci. 2015 Oct 21;16:68. doi: 10.1186/s12868-015-0209-8 (PMC4618870; doi:10.1186/s12868-015-0209-8)
Supplement: Supplementary file 6 — 10.1186/s12868-015-0209-8 Supplemental Methods and Results. [file 12868_2015_209_MOESM6_ESM.docx]

**Additional file 6: Supplemental Methods and Results**

**Behavior heatmaps:** Dynamic social behaviors between two mice during a resident-intruder paradigm were visualized in a 'behavior heatmap'. The behavior heatmap is a graphical representation of five identifiable behaviors (aggression, social investigations or sniffing, digging, grooming, and sitting or crouching in the corner) of each mouse over time. The behavior heatmaps show that there was a high degree of similarity in behavioral patterns within each group of mice whether they were residents or intruders. For example, the first heatmap in Additional file 1: Figure S1A (WT resident behavior is scored in the presence of a WT intruder) shows predominantly green pixels (sniffing/social investigations) at the start of the test. After about 15-20 s the color yellow (digging) becomes more abundant. The second heatmap (*Mras*^−^*^/^*^−^ resident scored in the presence of an *Mras*^−^*^/^*^−^ intruder) also starts with an abundance of green pixels (sniffing). However, in contrast to WT/WT resident/intruder pairs, green remains the predominant color for about four min., which means that most *Mras*^−^*^/^*^−^ residents rarely engaged in digging behavior but continued to investigate the intruders with only short interruptions. We noted that such long periods of sniffing often preceded the first aggressive attack (Additional file 1: Figure S1A shows this in three of six pairings where aggression occurred during the first five min. of the test) or a mating attempt with a female (not shown). Intruder behavior was very similar in both WT and *Mras*^−^*^/^*^−^ males regardless of the type of resident they were paired with: Intruders investigated the residents for about 30 s before exhibiting mixed behaviors with digging, social investigations, and grooming. The similarities in the patterns of observable behavior over time within each group of mice, as visualized by the heatmaps, suggest that it is unlikely that the respective behaviors were displayed randomly. However, the heatmaps particularly highlight differences between behaviors exhibited by WT and *Mras*^−^*^/^*^−^ males in their roles as residents, as described in the main Results section for Fig. 1.

**SDS gel electrophoresis and Western blots:** Mouse tissues were placed in cold lysis buffer (1 % NP-40, 200 mM NaCl, 50 mM Tris pH 7.5, 5 mM MgCl_2_, 15 % glycerol) with protease inhibitors (Roche). Soft tissues (such as brain or bone marrow) were lysed by pipetting up and down, tougher tissues (such as bladder or heart) were homogenized with an electric tissue grinder. After 15 min. in lysis buffer and removal of debris by centrifugation, 50 µg of lysate was loaded onto SDS gels. Western blots were developed with anti-M-Ras (2 µg/mL) and anti-goat IgG HRP-labeled secondary antibodies (1:2,000; both from R&D Systems) and chemiluminescent substrate (Pierce). For the analysis of urinary proteins, 2 µL of randomly collected urine from each mouse was loaded onto a 15 % SDS gel. The gel was stained with Coomassie to visualize proteins.

**Secondary neurosphere growth:** Primary NS obtained at DIV7 were gently mechanically dissociated and cells passed through a 40 µm cell strainer. Cells were seeded into 96-well plates at 1,000 cells per well in NeuroCult NSC Basal medium with Proliferation Supplement in the presence of varying concentrations of EGF (all from StemCell Technologies) and grown into secondary NS for six days. WST-1 reagent (Roche) was used to estimate cell growth by measuring metabolic activity. The experiment was performed three times.

**Basic Olfaction:** Male mice (3-6 months old) were first habituated by placing them into a clean cage containing fresh bedding for five min. on three consecutive days. On the fourth day, one Kellogg’s froot loop (random color) was buried in the bedding near the center of cage. The time to the first stop and sniff in the froot loop area was recorded. WT: n = 10; *Mras*^−^*^/^*^−^: n = 9.

**Water maze:** Mice (males and females, three months old, n = 10 per group) were first habituated to the pool (filled with water with some skim milk powder mixed in; 110 cm diameter) and tested for their ability to learn to swim to a cued goal, a submerged but flagged platform with the flag visible and extending above the water. Each mouse was given eight runs, over two consecutive days, of up to 60 s to swim to and climb onto the platform. If the mouse did not reach the platform in 60 s, it was guided to it. During the habituation phase we confirmed that *Mras*^−^*^/^*^−^ mice were not physically impaired to swim or to climb onto the platform. A total of 20 trial sessions were conducted on the following five days, with of four daily trials. Three quadrants of the pool were used as semi-random start locations, the fourth quadrant contained the hidden platform but without the flag. The platform remained stationary in the same quadrant during all trial sessions. If a mouse failed to find the hidden platform within 60 s, it was guided to it. The interval between daily trial sessions was ~ 15 min. The latency to climb onto the platform was scored. Out of four daily training sessions, we excluded the worst run for each day and averaged the time to reach the platform. Results were analyzed by two-way ANOVA. Significant interactions between genotype and trial number were detected for both males (*p*= 0.0045) and females (*p*= 0.0267). To assess reference memory, a probe trial was given 24 h after the last acquisition day. The platform was removed and mice were given 30 s to search. The amount of time the mice spent in the quadrant where the platform used to be was scored.
